# Understanding repeated non-attendance in health services: a pilot analysis of administrative data and full study protocol for a national retrospective cohort

**DOI:** 10.1136/bmjopen-2016-014120

**Published:** 2017-02-14

**Authors:** Andrea E Williamson, David A Ellis, Philip Wilson, Ross McQueenie, Alex McConnachie

**Affiliations:** 1 General Practice and Primary Care, School of Medicine, Dentistry and Nursing, MVLS, University of Glasgow, Glasgow, UK; 2 Department of Psychology, University of Lancaster, Lancaster, UK; 3 Centre for Rural Health, Institute of Applied Health Sciences University of Aberdeen, Aberdeen, UK; 4 Robertson Centre for Biostatistics, Institute of Health and Wellbeing, MVLS University of Glasgow, Glasgow, UK

**Keywords:** missed appointments, data linkage, administrative data, health utilisation, health inequalities, social vulnerability

## Abstract

**Introduction:**

Understanding the causes of low engagement in healthcare is a pre-requisite for improving health services’ contribution to tackling health inequalities. Low engagement includes missing healthcare appointments. Serially (having a pattern of) missing general practice (GP) appointments may provide a risk marker for vulnerability and poorer health outcomes.

**Methods and analysis:**

A proof of concept pilot using GP appointment data and a focus group with GPs informed the development of missed appointment categories: patients can be classified based on the number of appointments missed each year. The full study, using a retrospective cohort design, will link routine health service and education data to determine the relationship between GP appointment attendance, health outcomes, healthcare usage, preventive health activity and social circumstances taking a life course approach and using data from the whole journey in the National Health Service (NHS) healthcare. 172 practices will be recruited (∼900 000 patients) across Scotland. The statistical analysis will focus on 2 key areas: factors that predict patients who serially miss appointments, and serial missed appointments as a predictor of future patient outcomes. Regression models will help understand how missed appointment patterns are associated with patient and practice characteristics. We shall identify key factors associated with serial missed appointments and potential interactions that might predict them.

**Ethics and dissemination:**

The results of the project will inform debates concerning how best to reduce non-attendance and increase patient engagement within healthcare systems. Significant non-academic beneficiaries include governments, policymakers and medical practitioners. Results will be disseminated via a combination of academic outputs (papers, conferences), social media and through collaborative public health/policy fora.

**Strengths and limitations of this study**

- This study will answer important questions relating to the health service component of tackling health inequalities.
- A large data set enables the researchers to follow the patients’ journey across the whole healthcare system.
- The study uses data security and linkage capabilities in a sensitive and robust manner.
- The study has a clear yet flexible data analysis plan using the expertise of a multidisciplinary research team.
- There are limitations of using administrative data from a range of data sources of variable data quality.

## Introduction

Tackling health inequalities is a global health priority^1^ and for health service provision to have an effective role, we should understand better the reasons behind, risks associated with and needs of patients who do not engage effectively with healthcare provision (even if it is free at the point of access); and tailor services better to meet those needs. There remains a lack of published work concerning repeated missed appointments, but previous research typically focuses on the financial costs associated with non-attendance. One estimate has placed the cost of missed UK general practice (GP; community-based family medicine) appointments at £150 million per year.^2^ Recent Scottish government data suggest that each missed hospital outpatient appointment costs National Health Services (NHS) Scotland £120.^3^ International data on costs to healthcare systems are sparse. In a complex adaptive system such as healthcare, the financial costs are contestable because clinicians will ‘catch up’ or get on with other care or administrative tasks. What is important are the costs of opportunities missed for improving patients’ health and the potential for substantial long-term savings to health systems.

Until now, studies investigating missed appointments have focused on single missed appointments or single disease areas and have indicated that they are associated with poorer health outcomes.^3–6^ Studies of single missed appointments have produced conflicting results when it comes to designing effective interventions that can increase attendance.^7–10^ This may be due to a reliance on small samples in disparate settings^11–15^ and conflation of patients who occasionally miss appointments with patients who have an established pattern of missing many.

The Health and Social Care Information Centre in England has recently published data about repeated missed appointments. From their analysis of recorded missed outpatient hospital appointments in England, 1 in 50 patients (65 590 of 3.5 million) who missed an appointment failed to attend three or more further appointments within 3 months.^16^

We hypothesise that repeated missed appointments reflect a pattern of behaviour. We use the term ‘serially’ missing appointments to reflect this pattern, which may be interrupted by attended appointments. Clinicians do report that patients who serially miss appointments (SMA) are of particular concern because they may have very poor health, may be socially disadvantaged and are high users of unscheduled care compared with patients who occasionally or never miss appointments.^17^

There is accumulating evidence that negative experiences in early life have pervasive consequences for health over the life course including ‘extensive evidence of a strong link between early adversity and a wide range of health-threatening behaviours’.^18^ This body of work therefore provides a conceptual framework for better understanding ‘chaotic lives’^19^ as an explanatory factor in health usage behaviours such as missed appointments, and introduces the possibility that serial missed appointments contribute to the inverse care law, which states that healthcare provision is least likely to be provided to those who need it most.^20^

In the UK, publicly funded GP provides almost universal coverage for the population and generates around 90% of health contacts. Appointment making is typically under the control of each patient directly. GP appointments therefore provide a sensible starting point for this study of health and other outcomes across patients’ life course. Subsequent results will also have relevance for global health systems where patients have direct access to a wider range of healthcare specialties.

Scotland has an established data linkage infrastructure which is under continuous development. This pathfinder study will, for the first time, link large GP data sets (including appointment data) with data from across patients’ whole journey through healthcare.

The overarching study question is: is serially missing GP appointments a risk marker for vulnerability and poorer health outcomes and thus a useful target for developing interventions to improve engagement in health promoting care across the health system?

### Aim and research questions

The overall aim of the study is to determine the relationship between GP appointment attendance, healthcare usage, preventive health activity, health outcomes and social circumstances taking a life course approach and using extracted health service and other relevant administrative data.

**Figure 1:**
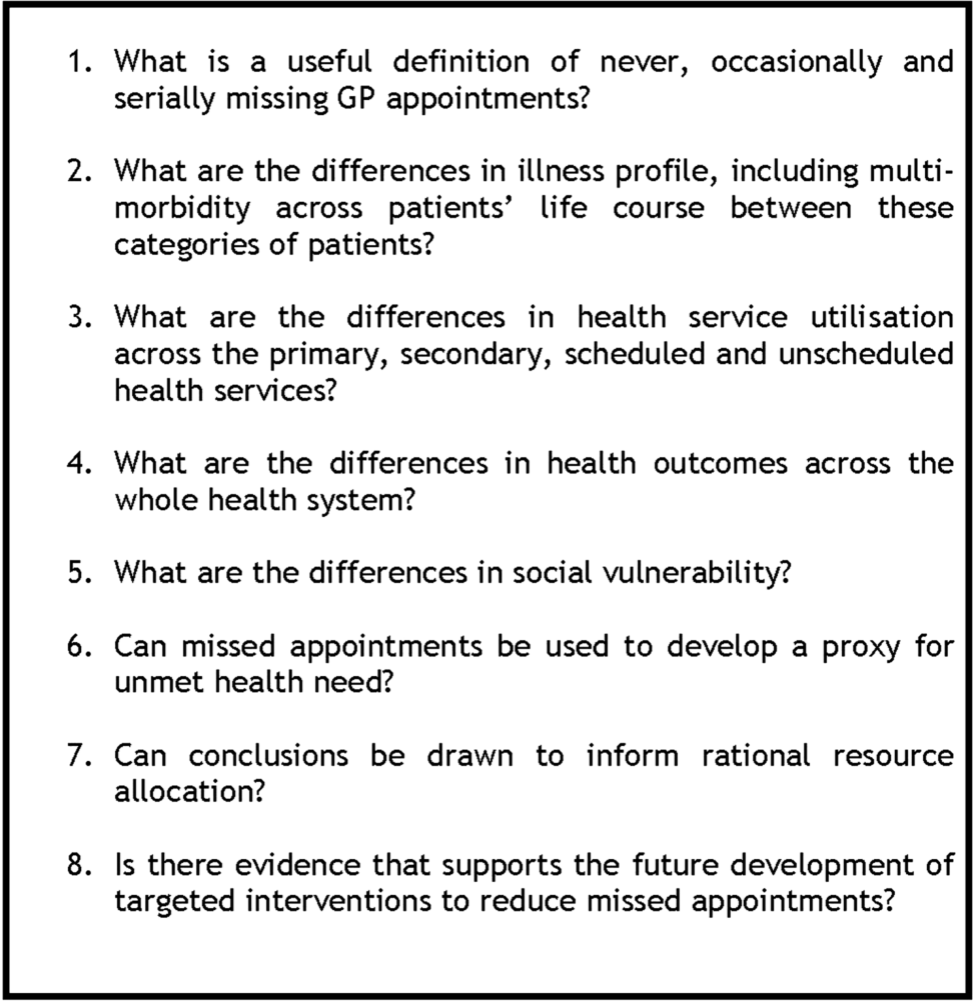
Study research questions.

A pilot study sought to answer the first research question described below (figure 1). The subsequent questions underpin the full research protocol which compares cohorts of Scottish patients (from birth to older age) who never, occasionally and serially miss GP appointments.

An introduction to the full study protocol is described, followed by the methods and results from a mixed-methods pilot study that informed the protocol. A description of protocol participants, data sources, variables and statistical analysis then follows.

## Methods and analysis

The full study protocol is for a retrospective cohort study of GP patient records linked with secondary care and education administrative records in Scotland.

The study started in July 2015 and will finish in December 2017. A pilot study was conducted between July and September 2015 which is described next. The cohort of 909 073 GP patient records for the full study was available in the National Safehaven from September 2016 and analysis of these data is underway. Permission to access education data is secured, and the outcome of linkage permissions for health data is not yet confirmed.

### Pilot study

The pilot study was separated into two subsections: a focus group to inform and refine definition development (research question 1) and a ‘proof of concept’ quantitative data analysis.

## Methods

### Focus group

A focus group was conducted in September 2015 with five GP participants. A focus group was judged the most appropriate method to use because we aimed to promote discussion of the topic such that participants would be able to compare and contrast their own experiences with others from a range of practice and professional experience settings.^21^ Linked to this was the aim of asking participants to make sense of and provide feedback on the presented pilot data. The GPs were a convenience, purposive sample based on two main principles. The first took into account the evidence surrounding single missed appointments. This describes missed appointments being more common in deprived urban practices. The sample therefore included GPs who worked in deprived and affluent urban areas and a practice with a significant rural practice population from Scotland. Second, the sample included the views of frontline GPs and GPs who had a range of strategic roles in practice development and GP management, locally and nationally. AEW and PW used their professional knowledge of GP networks and practice profiles to approach and recruit participants. Five out of 12 GPs contacted were able to attend the focus group. Each GP contacted reported that they felt this was an important topic worthy of attention. Barriers to attending were location of the focus group (conducted in Glasgow) and managing time away from other professional work. Online supplementary additional file 1 describes each participant's characteristics. Detailed information about participants’ practice characteristics was not collected. Three of the participants knew each other from their professional roles outside of clinical practice. AEW conducted the focus group and the analysis was conducted using Framework Analysis. Framework Analysis is a useful thematic analysis approach, especially when considering a focused topic like this one. Also in the context of being part of a larger mixed-methods study, epistemologically, its use was a good fit.^22^ DAE attended the focus group and presented initial results from the ‘proof of concept’ pilot (described next) for discussion. Online supplementary additional file 2 describes the topics covered in the focus group.

10.1136/bmjopen-2016-014120.supp1 supplementary additional file

10.1136/bmjopen-2016-014120.supp2 supplementary additional file

### Proof of concept

Research that uses GP appointment data has not previously been conducted using the clinical recording systems in the Scottish NHS. A proof of concept pilot study was undertaken using the NHS Trusted Third Party (TTP) Albasoft with 67 705 patient records to determine whether retrieving appointment data was feasible, to refine other data parameters and to inform the definition development as described in research question 1. An additional confidentiality control ensured that the research team did not know the identity of the recruited GP practices.

Online supplementary additional file 3 describes the definition and role of TTPs.

10.1136/bmjopen-2016-014120.supp3 supplementary additional file

**Figure 2:**
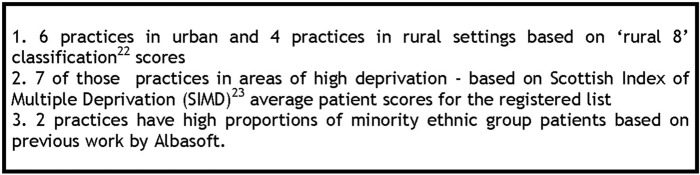
Pilot practice recruitment.

Albasoft purposively recruited 10 Scottish practices on our behalf with the practice characteristics illustrated in figure 2.

Data were cleaned and appointment rules applied to categorise appointments as attended or missed (Did Not Attend, DNA). Online supplementary additional file 4 describes this process. This was primarily based on the ‘in’ and ‘out’ time recorded for each appointment. If this was recorded as ‘0’, then the appointment was classified as DNA. For each patient, the total number of appointments made during the 3-year period was calculated, as well as the number and percentage of appointments missed. Appointments that were recorded incorrectly in the system were removed at this stage. These included appointments where administrative records had remained open for over 24 hours, making it difficult to confirm that these were genuine appointments and not administrative errors. The pilot appointment rules are set out in table 1 below.

**Table 1:** Rules to identify genuine appointments

Data description	Reason for removal
Total appointment time <0 min	Record open for more than 24 hours
Total waiting time <0 min	Record open for more than 24 hours
Appointment <2 min	Not a medical appointment
Administrator slot	Not a medical appointment

10.1136/bmjopen-2016-014120.supp4 supplementary additional file

## Results

### Focus group

Focus group participants reported making clear distinctions between patients who occasionally miss appointments and those who miss many. Patients who occasionally miss appointments do so because a crisis or another understandable circumstance has arisen; patients who SMA, described as missing more than two or three appointments, can be easily identified by GPs. They were described as tending to have mental health, addiction and/or social issues. They were described as high risk or vulnerable with concerns about their wider family. Patients who SMA were viewed as being different from the general GP population and being more likely to have lifestyles associated with housing instability, money problems and a panicked lifestyle (P2). Patients who SMA were also described as being unable to manage GPs’ expectation of them and fit into GPs’ predetermined slots. “there’s the occasional DNA which are quite normal and often those are quite significant [in total numbers for the practice] but the serial people I think that’s a reflection of the chaos in their life whether that's you know- mental health or issues with the social functioning- and inability to manage our expectation of them- to fit into our pre-defined slots” (P5).

All participants agreed with that view. However, one participant also considered that not all patients who SMA can be viewed as high risk; that instead some patients do not value free healthcare. It was reported that some patients who SMA go on to book another appointment the next day; “I don't think it’s the value of the GP- I think it’s the value of that appointment- I think the fact that it's, if you don't miss it, if you miss it is no big deal you just make another one” (P4).

Missed appointments were viewed as being more prevalent in practices in deprived settings, but occurred in affluent areas too. In the affluent setting, they were important for a minority of marginalised, isolated patients with the same profile as described above who were viewed as living ‘chaotic’ lives.

Practices represented in the focus group do not have protocols for managing patients who SMA because response is dependent on the patient's context. GPs understood that SMAs usually mean patients with complex needs with workload implications for the practice. Strategies described were varied, including allowing patients only to book on the day; “my impression is that deprived practices have a much higher percentage of on the day appointments because they skew it towards people that don't attend” (P3), seeing the patient when they walk in, or the GP booking the follow-up appointment for the patient—a relationship building strategy. This could still lead to patients missing an appointment, even just a couple of hours after it was made. It was reported that some practices do remove patients from their list for SMA and this created tension with other practices.

**Figure 3:**
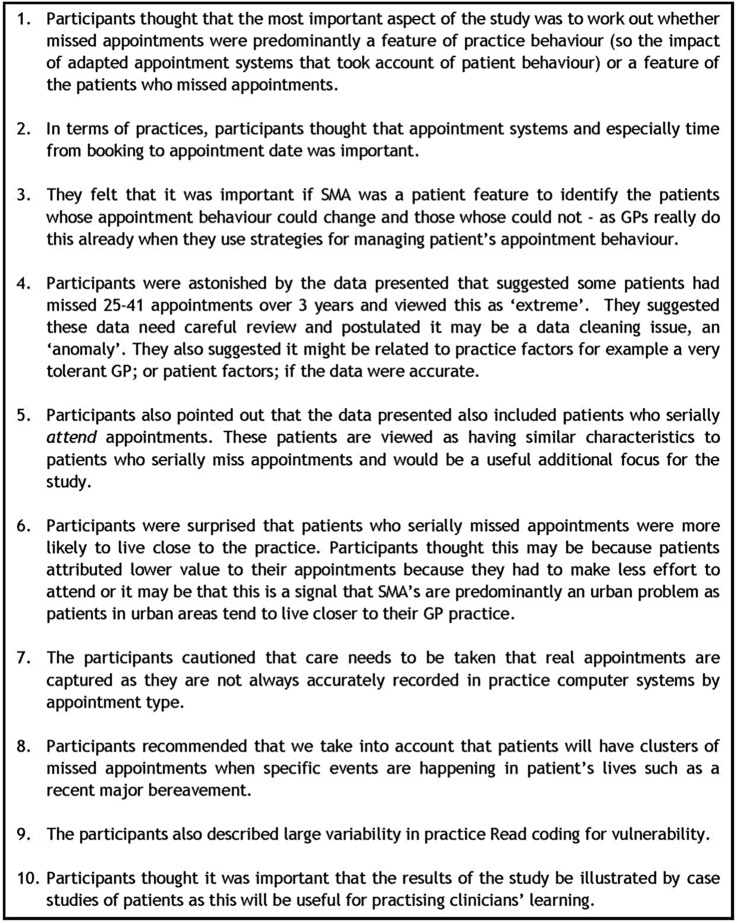
Focus group recommendations for the full study design.

The focus group was also asked to comment on the results from the proof of concept initial data and they made recommendations about the full study design described in figure 3.

### Proof of concept

A pilot analysis of 67 705 patient records showed that while just over 60% of our sample missed no appointments, over 30% missed one or more appointment during the 3-year period with nearly 10% of patients missing three or more appointments.

Assuming that our final sample provides a similar distribution, we will classify patients based on the number of appointments missed as follows:

- *Never* missed appointments: 0 per year average over a 3-year period.
- *Low* missed appointments: <1 per year average over a 3-year period.
- *Medium* missed appointments: 1–2 per year average over a 3-year period.
- *High* missed appointments: >2 per year average over a 3-year period.

Our sampling both in the pilot data stage and the final full study sample was conducted such that we were likely to get a representative sample of Scottish patients and practices. Since our pilot sample was large, it is appropriate to assume that this will scale up accordingly for the full study. The distribution of missed appointments also suggested useful categories based on integer numbers of missed appointments per year. This will be helpful for policy and clinical stakeholders.

## Full study protocol

### Participants and study size

**Figure 4:**
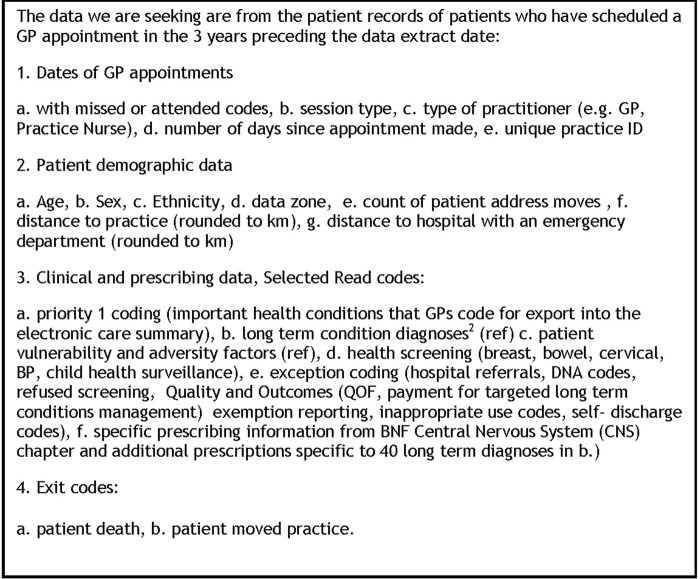
Information request sent to target practices.

Our target recruitment of GP practices seeks to ensure that a spread of urban and rural practices, affluent and practices characterised by serving areas of blanket high socioeconomic (Deep End) deprivation. The information request made to practices can be viewed in figure 4.

### Data sources and variables

#### GP data

The TTP has recruited the practices on the study team's behalf and will undertake some specific data aggregation before transferring the data securely to the National Safehaven for analysis. ‘Safehavens are specialised, secure environments supported by trained, specialist staff where data in electronic patient records can be processed and linked with other health data (and/or non-health-related data) and made available for analysis to facilitate research while protecting patient identity and privacy’.^23^ These are: calculating urban rural classification, Scottish Index of Multiple Deprivation (SIMD) decile, categorising ethnicity into ‘non-black and minority ethnicity (BME)’, ‘visibly BME’, and ‘non-visible BME’ and rounding travel distance to practice/emergency department for each patient record to the nearest kilometre. Once in a Safehaven, additional steps will be taken to ensure that acceptable anonymisation principles are being applied, especially in relation to reporting of sensitive social vulnerability data and reporting of rare conditions.

A new data file containing the appointment history for each patient record will be generated, which will be merged with individual patient information (online supplementary additional file 4 describes this process based on our pilot data set).

#### Appointment validation and categorisation

**Figure 5:**
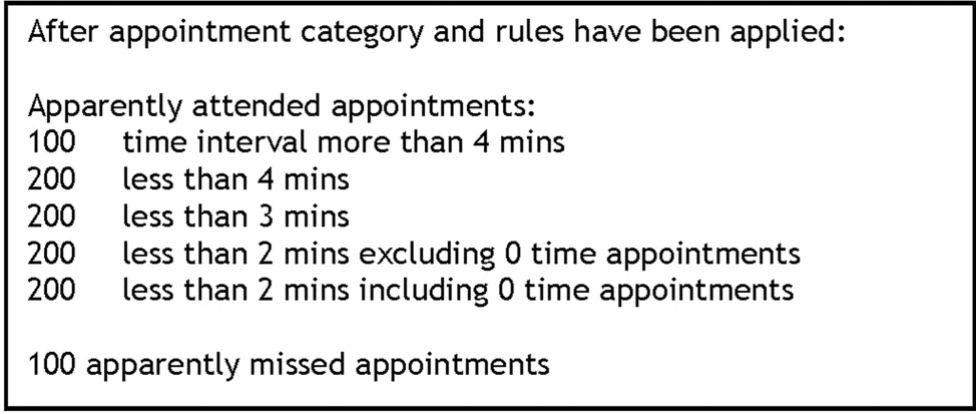
Random sample of GP appointments for validation and sensitivity analysis.

Each appointment will be coded based on session type recorded by the practice (eg, book on day appointments, or immunisation clinic) and then further by professional type (eg, GP partner, practice nurse). These descriptions are determined by individual practices, so categorisation will be conducted by the GPs in the research team. The appointment rules set out in the pilot study will be applied. A sensitivity analysis based on the time the appointment takes will then also be conducted by comparing a random sample of patient appointments as described in figure 5.

The appointment rules will be refined based on this. The time interval cut-off for apparently attended appointments will be determined by using the time interval that most accurately matches to actual attended appointments. Slots designated non-face-to-face appointments will then be removed leaving only attended and non-attended face-to-face appointments. The appointment categories described from the pilot study regarding non-attendance for all patients will then be applied to the yearly average number of missed appointments over the 3-year period to generate the four categories of patients for further analysis. Using an average over 3 years takes account of what is recognised in the frequent attenders (rather than non-attenders) literature that patients’ appointment behaviour may vary over time in relation to illness episodes or social crises.^24^

#### Health and education data linkage

**Figure 6:**
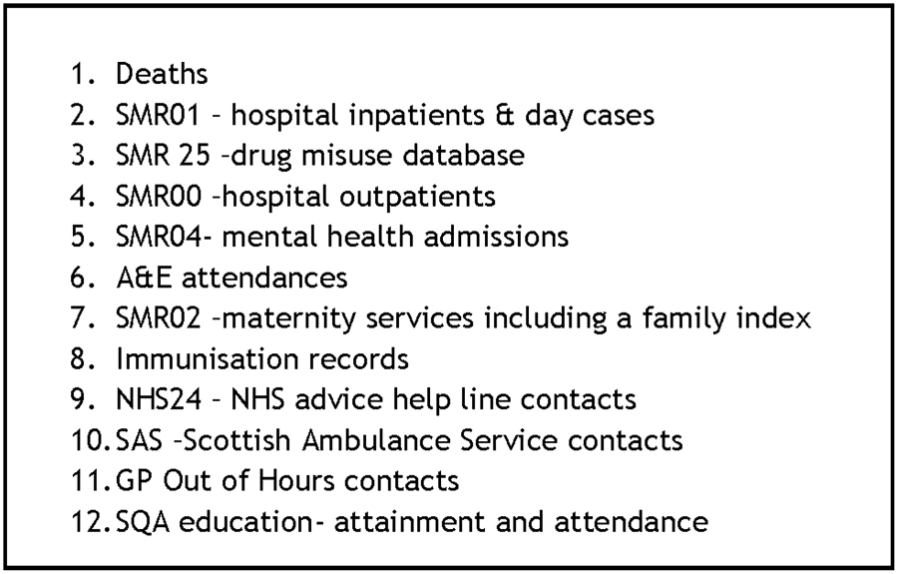
Proposed data sets for linkage with GP data. A&E, accident and emergency; GP, general practitioner; NHS, National Health Service; SMR, Scottish Morbidity Record.

Linkage will be conducted as access permissions and data sets become available (figure 6). Each administrative data source is available for different time periods (eg, hospital inpatients since 1981 and education outcomes since 2002) and this will be made explicit when interpreting the results. The TTP will provide the Safehaven indexing team with a file containing the GP data set Community Health Index (CHI) number and other patient identifiers. Every patient in the Scottish NHS has a CHI number, a unique identifier that is used as such across all NHS in Scotland. This forms the cohort for the study. All data providers will supply identifiers to be probability matched to the study cohort by the Safehaven linkage team (based on the CHI number and using other patient identifiers probabilistically for the small number of records where it is anticipated that CHI will be missing), who will return a set of unique index numbers for those individuals successfully matched to the study cohort; each data provider will receive a different set of unique index numbers, and will use these index numbers as the basis of their data extract. Each data extract will be submitted to the Safehaven linkage team, which will replace the different index numbers with a common number across all files. This common number is the unique patient identifier that the research team will work from during analysis.

### Bias

#### Accounting for patient turnover

This study seeks to ensure the inclusion of patients who are marginalised and who are often missing from health service studies. There is evidence of overlap between patients who miss appointments and those who are removed from practice lists,^25^ a recognition of the impact that geographical boundary areas have on patients who move around,^26^ notwithstanding the gap in the literature about registration interruptions for patients who may go to prison or patients who remain unregistered once they are removed from GP practice lists. We will therefore summarise the numbers of patients joining and/or leaving their practice during the study period, with reasons where this information is available. We will seek to provide a full analysis of the data available for these patients and compare these with the patients who are registered for the 3-year study period. Patients who are not registered with participating practices, and are being seen as ‘temporary residents’ by these practices, are excluded from the study. This is because these patients’ full clinical record is held by their registered GP, so very limited information is available. Temporary residents tend to be people on holiday in the practice area but will include some people who would be considered marginalised.

### Statistical methods

Our statistical analysis is based on the study being a retrospective cohort study. We will focus on two key areas: predictors of high rates of serial missed appointments, and serial missed appointments as a predictor of future patient outcomes.

Patient characteristics and practice characteristics may be associated with high rates of serial missed appointments. Analyses will initially be descriptive, summarising the rate of missed appointments in relation to the other factors recorded at the point of entry to the study. Associations with patient characteristics will be assessed as a whole, and in relation to different types of practices (eg, separately in rural and urban practices). Subsequently, we will build regression models (Poisson or negative binomial),^27^ to help understand how our categories of missed appointments are associated with patient and practice characteristics.

When considering other outcomes in relation to serial missed appointments, the missed appointment rate category (none, <1, 1–2 or >2 per year) will be the predictor variable. Appropriate regression models, according to the outcome, will be used to assess whether any associations with serial missed appointment rates are independent of other patient-level or practice-level factors. Conflicting interactions will be controlled for by using an ‘offset term’ in our negative binomial model which accounts for number of appointments made or any other relevant factors.

We also plan to measure relevant quantitative variables (described next) recorded *during* the study interval associated with having a lot of missed appointments. We will explore whether these *differ* from the predictive factors already recorded at entry to the study.

### Quantitative variables

The following potential predictors of frequent non-attendance will be analysed:

- Demographics;
- Patients’ age, gender, minority ethnic group status (where available), deprivation decile, rural/urban split, number of address moves, distance lived from GP practice and distance from nearest accident and emergency (A&E) will be explored;
- Health conditions.

Health conditions will be reported using separate categories:

1. The incidence of multimorbidity calculated from extracted Read codes based on previous counts in Scotland.^28^
2. Descriptions of health conditions based on the priority 1 Read codes that GP practices in Scotland use to populate patients’ key information summaries for GP out of hours services. This is novel work as a coding structure has not previously been applied to these Read codes. Read codes are the clinical coding system used in UK GP to record clinical and administrative activity and diagnoses.
3. A count of psychotropic medicine prescriptions based on the British National Formulary. This is in order to describe levels of psychological morbidity that are not captured by diagnostic criteria.
4. These variables will then be compared with the International Classification of Diseases (ICD) 10 coding data from patients’ secondary care linked data compiled from hospital admissions and outpatient attendances. Diagnostic data from emergency department attendance was deemed not of sufficient quality to use.

#### Social vulnerability

One aspect of this study which is particularly ground-breaking is our investigation of retrievable information about patients’ social vulnerability. The Adverse Childhood Experiences (ACE) questionnaire^29^ will be used as a template to match its nine descriptors of adversity to relevant Read codes in the patient's GP record. In addition, coding that maps the consequences of ACE will be analysed. A recent quantitative evaluation of Severe and Multiple Disadvantage will also be matched to GP Read codes. This examines the overlap of patients being homeless, in substance misuse services or in prison over the preceding year.^30^ Further, an exploration of additional Read codes that describe social vulnerability will be mapped. An anonymised text search linked to Read codes from the data set will provide additional information about social vulnerability as it is recorded in the free-text portion of GP records. Taken together, these will provide the first research evidence about the breadth and depth of social vulnerability recording by GPs.

#### Healthcare usage

Read coding in relation to cervical, breast and bowel screening attendance will be retrieved in addition to the proportion of patients who have had their blood pressure checked and have participated in child health surveillance and vaccination programmes across the life course. A subanalysis of usage of practice nurse and other healthcare professionals’ appointments in the practice will also be conducted and include an exploration of the relationship between attending all primary care appointments and categories of non-attendance. This is because data from the GP focus group suggested that there is an overlap between patients who are serial non-attenders and patients who are very frequent attenders. We will therefore consider the rate of attending appointments as a potential predictor of the rate of non-attendance. Referrals that GPs make into other primary and secondary care services will also be analysed. Outpatient attendances, hospital admissions and usage of emergency departments, NHS 24 triage, GP out of hours and ambulance services will also be analysed when linked data become available with a specific focus on how this relates to unmet need, for example, how might GP appointment category relate to patterns of other healthcare usage between scheduled and unscheduled secondary care use?

#### Healthcare engagement

An analysis of GP Read codes and linked secondary care data will be carried out in the following categories:

1. Patients not attending primary and secondary care appointments;
2. Patients refusing screening;
3. Patients being exception-reported (ie, excluded from the denominator population) from the Quality and Outcomes Framework (QOF) system for performance measurement in GP;
4. Practices’ measures of non-engagement with care for long-term conditions;
5. Patients taking ‘irregular discharge’ from hospital (when patients leave against medical advice);
6. Patients not waiting to be seen in emergency departments.

#### Family linkage

Diagnoses of children who are able to be linked through family linkage will be analysed based on their mother's appointment category. This is contingent on the child being included in the practice study population.

#### Education data

Attendance at school, exclusion from school and educational attainment when leaving school will be made with approximately a sixth of our patient cohort for whom linked education data are available. This has the potential to inform future planning around earlier interventions to reduce serial missed appointments.

#### Practice-level data

Each patient record will be allocated a unique practice ID enabling the research team to analyse each patient record output clustered by practice. This will be the proportion of patients aged over 75, by ethnicity (proportion BME), patient rurality, patient level of deprivation decile, patient distance to practice, distance to A&E appointments offered/engaged, days from when appointment is made, multimorbidity count, ACE score more than 4, Severe and Multiple Disadvantage score, hospital referrals and proportion of each appointment category by practice. These analyses and output will be refined as the study proceeds taking patient-level findings and multilevel modelling that characterise the respective contributions of practice-level and individual-level factors to missed appointment patterns.

#### Health outcomes

Mortality data regarding date and cause of death will be used from GP and linked data. This will sit alongside additional linked obstetric outcomes (from the Scottish Birth Record) for relevant women. Table 2 summarises the study quantitative variables.

**Table 2:** Summary of quantitative categories and variables

Data categories	Variables
Patient demographics	Age Sex Ethnicity Count of address moves Distance to practice Distance to A&E SIMD decile Rural8 score
Health conditions	Multimorbidity count Priority 1 read codes Psychotropic medication prescribing (BNF chapter) Secondary healthcare diagnoses (inpatient and outpatient)
Social vulnerability	Adverse Childhood Experiences Severe and Multiple Disadvantage General social vulnerability coding frame
Healthcare usage	Breast screening Bowel screening Cervical screening BP checked Child health surveillance Vaccinations Practice nurse appointments Other healthcare professional appointments Primary care attendance distribution Hospital referrals Outpatient attendances Hospital admissions Emergency departments attendance NHS 24 triage GP out of hours Ambulance services callouts
Healthcare engagement	DNA codes Refused screening QOF exempt Inappropriate use codes Self-discharge codes
Study exit	Patient death Patient moved practice
Family linkage	Secondary healthcare linkage with mother and child
Education data	School attendance School exclusion School attainment
Health outcomes	Cause of death
GP practice characteristics	Practice list size Patient age distribution Ethnicity category distribution Patient rural8 score distribution Patient SIMD score distribution Patient distance to practice distribution Patient distance to A&E distribution Number of appointments offered/patients engaged past 3 years distribution Number of days since appointments made distribution Patient multimorbidity score distribution Patient ACE score distribution Patient SMD score distribution Patient hospital referrals distribution Primary care attendance pattern distribution

A&E, accident and emergency; ACE, Adverse Childhood Experiences; BNF, British National Formulary; BP, blood pressure; DNA, Did Not Attend; GP, general practitioner; NHS, National Health Service; QOF, Quality and Outcomes Framework; SMID, Scottish Index of Multiple Deprivation; SMR, Scottish Morbidity Record, SQA, Scottish Qualification Authority; SMD Severe and Multiple Disadvantage.

## Ethics and dissemination

This pathfinder linkage retrospective cohort study is necessarily complex in design and implementation because, although cross-sectional, it seeks to take a life course approach and follow the patients’ journey through the healthcare system. Careful attention and significant resource has been devoted to the consideration of patient privacy and confidentiality. This has been integrated throughout the design of the study alongside the necessary data access and handling permissions. Additionally, a study of this nature, which involves stakeholders across the NHS and other public services, requires a flexible time frame to allow access to raw data and to share findings between members of the research team based in several institutions. The proof of concept pilot did not require ethical approval because it was considered service evaluation with the agreement that we would not publish any results about the practices which took part. A letter of comfort was obtained from the West of Scotland NHS Ethics Committee and the MVLS Ethics Committee confirming that the full study did not need health service ethics permissions.

Owing to the sensitive nature of administrative data from the NHS and public education system in Scotland, the data sets generated and/or analysed during the current study will not be publicly available. They have been made available to the research team under controlled access and strictly for the purposes of this research study only. Summary data, at the level of disclosure checked output from the National Safehaven and statistical code, can be requested from the corresponding author on reasonable request.

### Planned outputs

Alongside peer-reviewed academic papers reporting the findings described above, the following additional outputs are planned.

#### Data visualisation

Several web pages will be built to sit alongside key results. This will allow for the rapid construction of interactive data visualisations which will be created using ‘Shiny’,^31^ a web application framework for R which is the statistical software used for the study analysis. A simple platform will allow researchers and collaborators to interact with the analyses in real time and generate custom tables and graphs as required. It can also provide non-experts with access to simple and complex statistical analysis using a point-and-click interface. This will not rely on raw data and will simply pull information from the summary descriptive analyses.

#### Case studies

We also intend to use case studies to develop and illustrate our findings throughout the course of all our analyses. For example, we will be able to identify typical patient profiles of those who appear to miss many appointments in a very short period of time and compare these events with short-term and long-term health outcomes.

## Conclusion

We shall identify key factors associated with serial missed appointments ranked in order of importance as described above, but given the large sample size, we shall also be able to consider potential interactions that might predict serially missed appointments.

Finally, this approach also explores the theory that low engagement with healthcare should be viewed as a health harming behaviour, and will inform the debate about tackling health inequalities at the health service delivery level. Moving from theory into application, the results will allow us to better understand and develop future interventions to reduce serial missed appointments.
