# Supplementary material for: Understanding repeated non-attendance in health services: a pilot analysis of administrative data and full study protocol for a national retrospective cohort
Source: BMJ Open. 2017 Feb 14;7(2):e014120. doi: 10.1136/bmjopen-2016-014120 (PMC5319001; doi:10.1136/bmjopen-2016-014120)
Supplement: supplementary additional file [file bmjopen-2016-014120supp1.pdf]

Additional file 1: GP focus group participant characteristics

Participant characteristics	Practice setting	Other work roles
• 4 male and 1 female GP<br>• All aged 40-55 years old	• 3 high urban deprivation<br>• 1 urban high affluence<br>• 1 mixed semi-rural with pocket deprivation	• 1 clinical director of a Health and Social Care Partnership<br>• 1 Local Medical Committee member<br>• 1 clinical lead for a national innovation project<br>• 2 with strategic Royal College of General Practitioner roles<br>• 2 members of the 'GPs at the Deep End' steering group
