# Supplementary material for: Understanding repeated non-attendance in health services: a pilot analysis of administrative data and full study protocol for a national retrospective cohort
Source: BMJ Open. 2017 Feb 14;7(2):e014120. doi: 10.1136/bmjopen-2016-014120 (PMC5319001; doi:10.1136/bmjopen-2016-014120)
Supplement: supplementary additional file [file bmjopen-2016-014120supp2.pdf]

Additional file 2: GP Focus Group Interview Schedule

Introductions:

Name, how long in clinical practice, time in your job, brief description of practice setting.

A priori knowledge/experience of missed appointments

Are missed appointments important? If so why? If not why not?

Can a distinction be made between patients who occasionally miss and those who serially miss GP appointments? If so what are those distinctions? Are they important?

How do you make that distinction in clinical practice? (probe distinctions between individuals and practice settings)

What does it mean for you, your practice and patients? Specifically patients who serially miss?

Present proof of concept provisional data (data cut offs, patient profiles)

What does this data tell us about the issue of serial missed appointments?

What are the obvious things it tells us? What are the surprises? Why?

Do you think it misses important aspects of what you think about the issue? Why might that be?

If we present these options about what a definition of a patient who serially missed appointments compared to one who occasionally does, which one do you think is most accurate? Why?

Is there more information that we should look for before deciding we have a definition? What should that be?

Conclusion

Finally, are there aspects of missed appointments and the definition development we have worked on today that we have not yet covered and you would like to tell us about?
