# Supplementary material for: Understanding repeated non-attendance in health services: a pilot analysis of administrative data and full study protocol for a national retrospective cohort
Source: BMJ Open. 2017 Feb 14;7(2):e014120. doi: 10.1136/bmjopen-2016-014120 (PMC5319001; doi:10.1136/bmjopen-2016-014120)
Supplement: supplementary additional file [file bmjopen-2016-014120supp3.pdf]

Additional file 3: Definition and role of TTPs

With the increasing demand for statistical, research and service planning information from primary care records a solution is required to reduce the exposure of patient and clinician information to the requesting organisations to a minimum. The recommended (Information Commissioners Office) method of achieving this is by using a trusted third party (TTP) as an intermediary between organisations, which significantly reduces the number of individuals with access to identifiable information. In this case the TTP's role is to provide the technical skills to extract the required information from the Data Controllers electronic records and process this into a form that is both fit for purpose and complies with principal 3 of the data protection act. This may require the removal/replacement of identifiers (anonymisation /pseudo-anonymisation) or the use of redaction techniques when only statistical information is required prior to release of information to the beneficiary.

A TTP is required to operate to strict guidelines as it may only processes data in accordance with instructions from the data controller and to a specification previously agreed by both data requestor and data provider. The TTP acts as a Data Processor on behalf of the Data Controller and abides by the principles defined in the data protection act. It is registered as a data processor with the ICO, provides a secure storage facility which operates procedural, physical and electronic access controls to protect the data it processes and has no specific interest in, not is affiliated to any organisation that has an interest in any data provided. Albasoft maintain a secure data processing and storage facility at the Centre for Health Science adjacent to Raigmore hospital in Inverness, this facility is solely hosted on the NHS network. No information is transferred out with the

NHS network. Its existing middleware platform Escro is an advanced practice based reporting system and is used to securely process data locally at the practice before transferring the results to their secure repository. Albasoft has an established track record as a TTP for the Scottish Therapeutics Utility and increasingly in supporting NHS research. In our study, Albasoft have established data sharing agreements with Scottish GP practices for computerised access to the GP practice data.
