# Supplementary figures and images for: Understanding repeated non-attendance in health services: a pilot analysis of administrative data and full study protocol for a national retrospective cohort
Source: BMJ Open. 2017 Feb 14;7(2):e014120. doi: 10.1136/bmjopen-2016-014120 (PMC5319001; doi:10.1136/bmjopen-2016-014120)

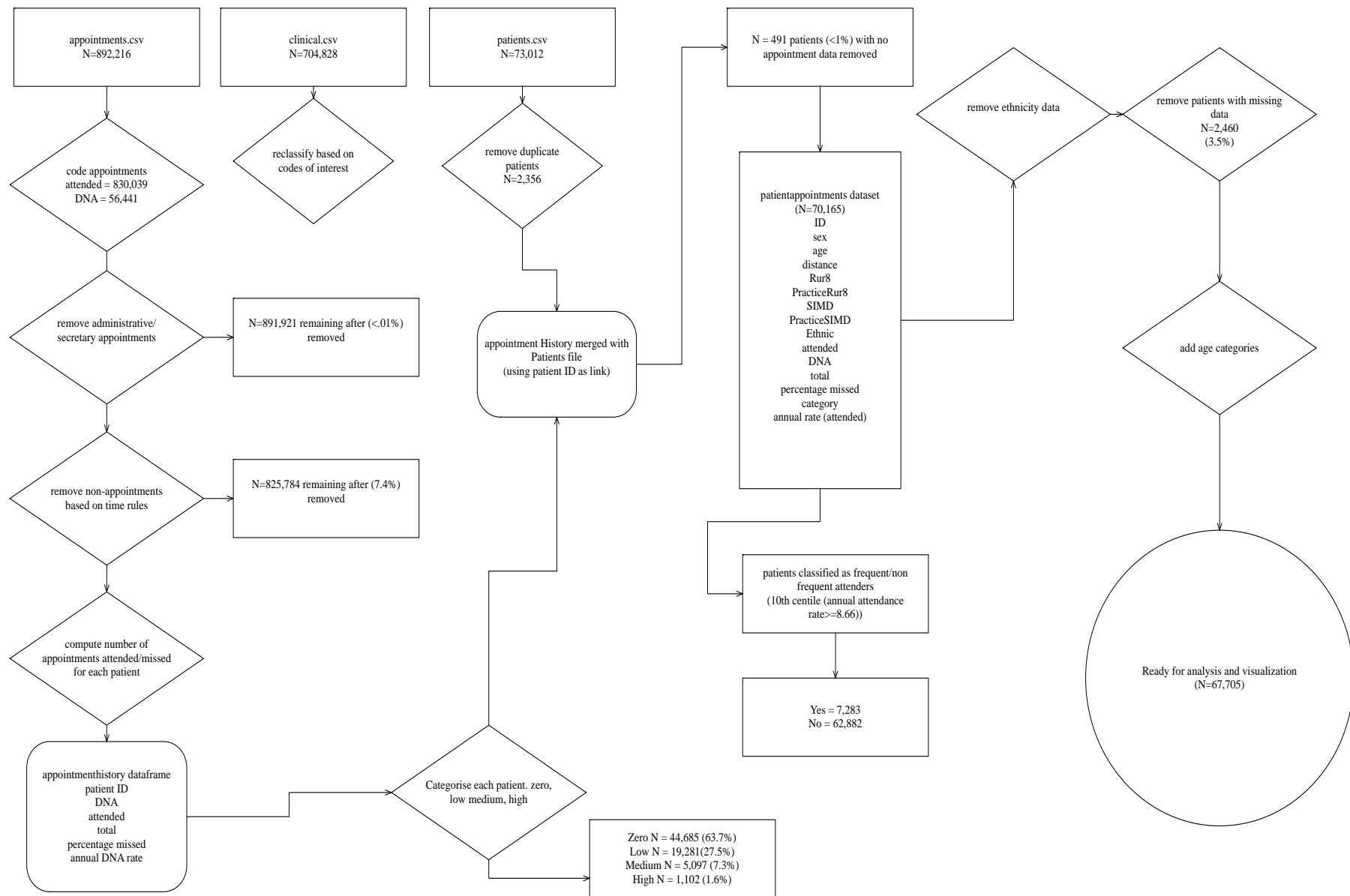

Supplement: supplementary additional file [file bmjopen-2016-014120supp4.pdf]
